# Historical Demography and Species Distribution Models Shed Light on Speciation in Primates of Northeast India

**DOI:** 10.1002/ece3.70968

**Published:** 2025-02-25

**Authors:** Mihir Trivedi, Kunal Arekar, Shivakumara Manu, Lukas F. K. Kuderna, Jeffrey Rogers, Kyle Kai‐How Farh, Tomas Marques Bonet, Govindhaswamy Umapathy

**Affiliations:** ^1^ Laboratory for the Conservation of Endangered Species CSIR‐Centre for Cellular and Molecular Biology Hyderabad India; ^2^ Centre for Ecological Sciences Indian Institute of Science Bangalore India; ^3^ Academy of Scientific and Innovative Research (AcSIR) Ghaziabad India; ^4^ Illumina, Inc. San Diego California USA; ^5^ Department of Molecular and Human Genetics, Human Genome Sequencing Center Baylor College of Medicine Houston Texas USA; ^6^ Institute of Evolutionary Biology (UPF‐CSIC), PRBB Barcelona Spain; ^7^ Institució Catalana de Recerca i Estudis Avançats (ICREA) and Universitat Pompeu Fabra Barcelona Spain

**Keywords:** climate change, distribution models, historical demography, Northeast India, primate, whole genome sequencing

## Abstract

Past climate change is one of the important factors influencing primate speciation. Populations of various species could have risen or declined in response to these climatic fluctuations. Northeast India harbors a rich diversity of primates, where such fluctuations can be implicated. Recent advances in climate modeling as well as genomic data analysis has paved the way for understanding how species accumulate at a particular geographic region. We utilized these methods to explore the primate diversity in this unique region in relation to past climate change. To ascertain the population level changes, we inferred the demographic history of nine species of primates found in Northeast India and compared it with species distribution models of Pliocene and Pleistocene period. Through this study, we are able to provide a detailed picture of how past climatic changes have resulted in the present species diversity and this mixture of species have either originated in the region or have dispersed from mainland Southeast Asia. We observe that effective population size has decreased for all the species, but distributions are different for all the four genera: Macaca, Trachypithecus, Hoolock and Nycticebus. It also gives an idea about how each species is affected differently by climate change, and why it should be given emphasis in framing species‐wise conservation models for future climate change.

## Graphical abstract

Northeast India's diverse geography has created numerous niches, promoting species evolution, with past climate change playing a significant role in primate speciation. Using climate modeling and genomic data, we studied the demographic history of nine primate species, revealing that climate fluctuations during the Pliocene and Pleistocene influenced current diversity, with some species originating locally and others dispersing from Southeast Asia. Our findings highlight the varying impacts of climate change on different genera, emphasizing the need for species‐specific conservation strategies.

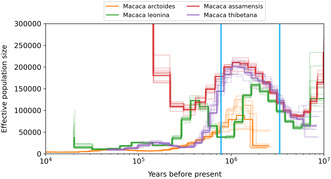

## 1 Introduction

Historical climate change is one of the major drivers in speciation (Hua and Wiens 2013; Carstens and Knowles 2007). Climate change has a drastic effect on adaptation of a population, and in the long term, also affects the process of speciation (Hoffmann and Sgró 2011; Hua and Wiens 2013; Parmesan 2006). Recent exacerbation of climate change have also documented this effect as many species have shifted their range to a more favorable habitat (Evans et al. 2020; Neumann et al. 2022; Poessel et al. 2022), change their body size (Sheridan and Bickford 2011), alter their reproductive physiology (Réale et al. 2003) and phenological delay (Lane et al. 2012). Even a small magnitude of change in climate, if persistent for long, can influence natural histories of species and their distribution in a particular area (Parmesan and Yohe 2003).

Climate change can give rise to a variety of selection pressure on a population, or on a species. For example, thermal stress produces directional selection on species which cannot regulate their body temperatures like porcelain crabs and lizards (Huey et al. 2009; Stillman 2002). A population can save itself from extinction during climatic change events through phenotypic plasticity or undergoing evolutionary adaptations or moving in a more suitable area (Crispo et al. 2010; Williams et al. 2008). Movement of a species involve range boundaries where the feedback between ecology and evolution is strongest, with high selection pressure and where processes like population bottlenecks are common (Hill, Griffiths, and Thomas 2011). Species' range shifts due to climatic fluctuations have occurred throughout the Earth's history with historical glacial cycles causing many such shifts (Beyer and Manica 2020; Davis and Shaw 2001; Jackson and Overpeck 2000). Climate change also plays a predominant role in processes like resource availability, propagule availability, competition, predation and so forth by disturbing established interactions or by giving rise to new interactions (Lavergne et al. 2010). Influence of climate on rates and patterns of speciation can be one of the important causes of present patterns of biodiversity, particularly on the high richness of tropical regions (Mittelbach et al. 2007).

The northeast region of India, a part of larger Indo–Burma diversity hotspot, is one of the most diverse habitats of India (Myers et al. 2000). Such rich biodiversity and endemism are possible due to the confluence of biogeographic regions like Indo—Malayan, Indo—Chinese and mainland India. Furthermore, with its variety of altitude ranging from Eastern Himalayas to plains and many valleys of Brahmaputra and Barak rivers, the region provides enough opportunities to a plethora of species to survive in an array of niches. This makes it a unique region to study the species' range dynamics and speciation. There are 14 species of primates found in the region (Ghosh et al. 2022; Talukdar et al. 2021). The wealth of resources in the northeast India has also attracted various challenges to its wildlife and fragile ecosystem. As a result of this, all the primate species in northeast India, with the exception of Rhesus macaque, are assessed to be Near Threatened or at a higher risk by IUCN Red List. Therefore, it is an urgent call to expedite our understanding of their evolution and provide appropriate recommendations for their conservation and management.

Many studies have investigated the effects of past climate change on evolution of various metazoans in different parts of the world, with scales ranging from centuries to millions of years (de Lafontaine et al. 2018; Nogués‐Bravo et al. 2018; Quintero and Wiens 2013; Smith, Betancourt, and Brown 1995; Theodoridis et al. 2020). Various studies have shown the impact of climate oscillations on primates (de Lima et al. 2019; Korstjens and Hillyer 2016; Reed and Fleagle 1995). In accordance with these studies, we investigated nine species from the region—which were part of the global Primate Genome Sequencing Initiative (Kuderna et al. 2023; Gao et al. 2023) – to elucidate the reasons for such diversity. We hypothesized that these species of primates in NE India show dramatic variation in the effective population size and their distributions in historical periods with climate fluctuations. Our approach included two separate dimensions: (1) to model their historic distribution by extrapolating current geographical locations; (2) to assess the demographic history of individual species. Demographic history will provide the fluctuations in effective population size which has been implicated in speciation events (Chen et al. 2020; Harvey, Singhal, and Rabosky 2019). For distribution modeling, we used layers from two epochs: Pliocene and Pleistocene. Pliocene, at 3.3 million years ago is the oldest period available for species distribution modeling and it also makes a suitable model system for future climate prediction scenarios (Tierney et al. 2020). Another mark at 787,000 years ago, is the oldest Pleistocene interglacial with almost identical conditions as present. We used these complementary approaches and incorporated previously published divergence details and cladogenetic events from the literature to investigate how the species' distribution in northeast India in Pliocene and Pleistocene relates with the effective population size and how this relationship can provide insights on the drivers of primate diversity in the northeast India.

## 2 Materials and Methods

For this study, we used two different, but harmonizing approaches of modeling historical distribution as well as estimating the historical effective population size of the nine species of primates. We used the data generated for Primate Genome Sequencing Initiative. Here in this section, we detail the sequencing, aligning to reference, MSMC2 and Maxent modeling, respectively.

### 2.1 Sampling, Library Preparation and Sequencing

The data was generated as part of the Primate Genome Sequencing Initiative (Kuderna et al. 2023). The species used in study are: *Macaca arctoides* , *M. leonine* , *M. assamensis* , *M. thibetana* , *Trachypithecus pileatus* , *T. phayrei* , *T. geei* , *Hoolock hoolock* and *Nycticebus bengalensis* . Briefly, we collected blood samples of nine primate species of Northeast India from captive animals in different zoos and rescue centres. It is worth to be noted that these animals were rescued from wild and not bred in captivity. We isolated high‐quality genomic DNA from the blood samples stored in vacutainers using the Qiagen Blood and tissue kit. We assessed the quality and quantity of genomic DNA using Agarose gel electrophoresis, Nanodrop, and Qubit 4. We used about 1ug of genomic DNA as input for library preparation using the Illumina Truseq DNA PCR‐free library preparation kit. We fragmented the genomic DNA to an average size of 350 bp using the Covaris Ultrasonicator. We then size selected the fragments between 220 and 550 bp using the AMPure XP beads. The fragments were then end‐repaired, dA tailed, and ligated with adaptors containing the IDT Unique dual indices for Illumina. We purified the libraries and verified them on the Fragment Analyzer and quantified on the Roche Lightcycler qPCR using the KAPA library quantification kit for Illumina. The libraries having good concentrations were then normalized and pooled together with other samples. We sequenced the pooled libraries in paired‐end mode (150 bp × 2) on the Illumina Novaseq 6000 platform using the S4 flow cell targeting about 100 Gb of sequencing data per sample corresponding to a coverage above 30× for an expected genome size of 3 Gb for primates.

All procedures were approved prior to blood sampling by the Internal Animal Ethics Committee of the CSIR‐Centre of Cellular and Molecular Biology, Hyderabad (approval number IAEC 07//2019 dated 06‐05‐2019). Blood sample collection from captive animals was authorized by the Principal Chief Conservator of Forests & Chief Wildlife Warden of the respective state forest departments, in compliance with the Wildlife Protection Act of India, 1972. Sample collection was performed by professionally trained veterinarians at the respective zoological facilities, ensuring minimal distress to the animals.

### 2.2 Quality Control, Read Mapping and Variant Calling

We demultiplexed the base call data using the unique dual indices of respective samples with the BCL2FASTQ utility provided by Illumina. We interleaved the forward and reverse reads with SEQTK (https://github.com/lh3/seqtk) and trimmed the residual adaptor sequences and low‐quality ends with CUTADAPT (Martin 2011). We discarded all the short fragments below 30 bp after trimming. We mapped the quality‐controlled reads on to the reference genome of taxonomically nearest species since the genome assemblies of our species of interest was not available. We used the *Macaca mullata* assembly (GCA_003339765.3, Mmul_10) for all the Macaques, *Rhinopithecus roxellana* assembly (GCA_007565055.1, ASM756505v1) for the *Trachypithecus* species, *Nomascus leucogenys* assembly (GCA_006542625.1, Asia_NLE_v1) for the *Hoolock* gibbon, and *Nycticebus pygmaeus* assembly (Primate Genome Sequencing Initiative, unpublished) for the slow loris (Table 1). We used BWA MEM (H. Li 2013) to map the reads to the respective reference genomes and used SAMTOOLS (Li et al. 2009a) to sort the alignments by coordinates, merge all the libraries of a given sample, and convert to the BAM format. Then we proceeded to polish these alignments. We then marked all the duplicate reads using the BIOBAMBAM suite (Tischler and Leonard 2014), added the read groups using the PICARD tools (http://broadinstitute.github.io/picard) and indexed the bam files for downstream processing. We used the GATK HaplotypeCaller and GenotypeGVCFs (Poplin et al. 2018) to call the variants on a per‐sample basis with single base resolution. We calculated the coverage of all sites and filtered out regions below 1/3rd of mode coverage or 2 times above the mode coverage of respective samples. We also filtered out the heterozygous sites having a support of less than 3 reads and used BCFTOOLS (Danecek et al. 2021) to filter the variants using the hard cut‐offs recommended by GATK best practices (DePristo et al. 2011).

**TABLE 1:** Number of individuals for each species. The “Reference species” shows the species on which mapping of the concerned “Species” was done.

S. No.	Species	Number of individuals	Reference species
1	*Macaca arctoides*	4	*Macaca mulatta*
2	*M. assamensis*	1	*M. mulatta*
3	*M. thibetana*	1	*M. mulatta*
4	*M. leonina*	5	*M. mulatta*
5	*Trachypithecus phayrei*	1	*Trachypithecus phayrei*
6	*T. pileatus*	1	*T. phayrei*
7	*T. geei*	6	*T. phayrei*
8	*Hoolock hoolock*	11	*Nomascus leucogenys*
9	*Nycticebus bengalensis*	4	*Nycticebus bengalensis*

### 2.3 Demographic History

Using coalescent‐based analysis with Multiple Sequentially Markovian Coalescent (MSMC2), which takes the whole genome sequences for the past population size inference (Schiffels and Wang 2020), we reconstructed the demographic history of each species. MSMC2 used the heterozygous sites for its algorithm and therefore it is important to make appropriate input files with MSMC tools package (https://github.com/stschiff/msmc‐tools). We created scaffold‐wise input files of all the individuals of each species that contained the positions of heterozygous sites and their distance from the last observed heterozygous site using the MSMC tools package. To account for any uncalled positions in the genome due to abnormal coverage, we provided callability masks for each sample. We ran the MSMC2 program for 20 iterations using the Baum‐Welch algorithm to maximize the likelihood. We used the default time segment patterning (1x2 + 25x1 + 1x2 + 1x3) with 32 time segments and 28 free parameters. In order to obtain the variance of the estimated coalescent rates, we generated 10 bootstrapped replicates per sample by creating 30 artificial chromosomes, each with 1 Mb randomly sampled blocks with replacement. MSMC2 provide scaled time and coalescent estimates, which needs to be converted to years and effective population size. We did this by using the most recent estimates of generation length and per‐generation mutation rates from IUCN Red list and literature (Table S1). Finally, we excluded the last infinite time segment and plotted the values of scaled time and effective population size to visualize the demographic history.

### 2.4 Paleo‐Distribution Modeling

#### 2.4.1 Data Collection

The occurrence data for the nine species was collected from the GBIF (Global Biodiversity Information Facility) database (see references for DOI of each dataset). For each species, we plotted these occurrence records on the map and included only those records which fell within the known distribution zones of the respective species, as described in by the IUCN. In some instances, the GBIF dataset had multiple data points for the same location (latitude and longitude coordinates). Therefore, in order to obtain a single occurrence record per location, we used the spThin package in R v4.0 (Aiello‐Lammens et al. 2015).

Further, we downloaded the bioclimatic variables from paleoclim.org (Brown et al. 2018), we downloaded the layers for three different climatic periods—current (1979–2013), Pleistocene MIS19, ca. 787 ka (thousand years ago) (Brown et al. 2018) and Pliocene M2, ca. 3.3 Ma (million year ago) (Dolan et al. 2015). Both the past layers, that is, the Pleistocene and Pliocene, contained only 14 bioclimatic layers—bio1, bio4, bio8, bio9, bio10, bio11, bio12, bio13, bio14, bio15, bio16, bio17, bio18, and bio19, because the remaining layers could not be created for these time periods, as mentioned in paleoclim.org (Table S5). Therefore, for the current time period as well, we used the same 14 layers and removed the rest. Spatial resolution of all the layers was 2.5 arcmins. Using ArcMap v10.2.2, all the bioclimatic variables were clipped for the region from 67.12°E to 124.12°E and from 12.6°S to 39.35°N. The clipped layers were exported into ASCII format for further use. These layers were then checked for multicollinearity using the correlation option in ENMtools v1.3 (Warren, Glor, and Turelli 2010). Correlation was checked using current layers.

#### 2.4.2 Variable Selection

For each of the nine species, a test run was performed using Maxent v3.4.3 (Phillips, Anderson, and Schapire 2006). Here, we used layers from only the current time period, all the 14 bioclimatic variables were used for this test run. Default values were used for the run except for the following modifications; feature type = Auto, RM (Regularization multiplier) = 1, maximum iterations = 5000, replicates = 10 and replicate type = subsample. After the analysis, only those variables were chosen whose percent contribution was greater than 1%. We selected variables with more than 1% as a heuristic approach to minimize the chances of overfitting and false positives. From these chosen variables, only the variables with the correlation (*R*) ≤ 0.75 were selected for the final analysis. Out of the two correlated variables, one variable was arbitrarily selected to be included in the analysis.

#### 2.4.3 Model Selection

The complexity of Maxent models is controlled by type of feature class (FC) and the value of regularization multiplier (RM) (Morales, Fernández, and Baca‐González 2017; Radosavljevic and Anderson 2014) which in turn controls the quality of the Maxent output (Shcheglovitova and Anderson 2013). Therefore, to find the best combination of FCs and RM value, we performed model selection. Maxent v3.4.3 has the following FCs—linear (L), quadratic (Q), product (P), hinge (H), threshold (T), and Auto (Phillips et al. 2017)—although ‘Auto’ itself is not a feature class but we included it in the analysis as we wanted to select the best combination of both the parameters mentioned above—whereas the default RM value is 1. We used 11 sets of FCs—H, L, LQ, LQH, LQP, LQPT, LQPTH, Q, QPT, QPTH, and T. These 11 sets resulted from either a single FC or a combination of multiple FCs. Then for each of these 11 FCs, we used four different RM values—0.5, 1, 2, and 5. In total, for each of the nine species, we tested 44 different models (model here is a combination of FC and RM value). The best model was selected (Table S2) by performing the model selection analysis in ENMTool v1.3 using the AICc criteria (Galante et al. 2018).

#### 2.4.4 Climate Suitability Analysis in Maxent

For all the 9 species of primates, the final analysis was performed in Maxent v3.4.3 using the following modifications; random test percentage was set to 25%, maximum number of background points was set to 10,000, the replicates were set to 10 and the replicate type was changed to sub‐sample. 5000 iterations were performed, Jackknife test was performed to estimate the contribution of each environmental variable. The FC type and RM values were different for each species and were selected based on the model selection analysis (Table S2). The output format was chosen as Cloglog. AUC values were examined to check for the predictive ability of the model built.

## 3 Results

### 3.1 MSMC2 Estimates

**FIGURE 1:**
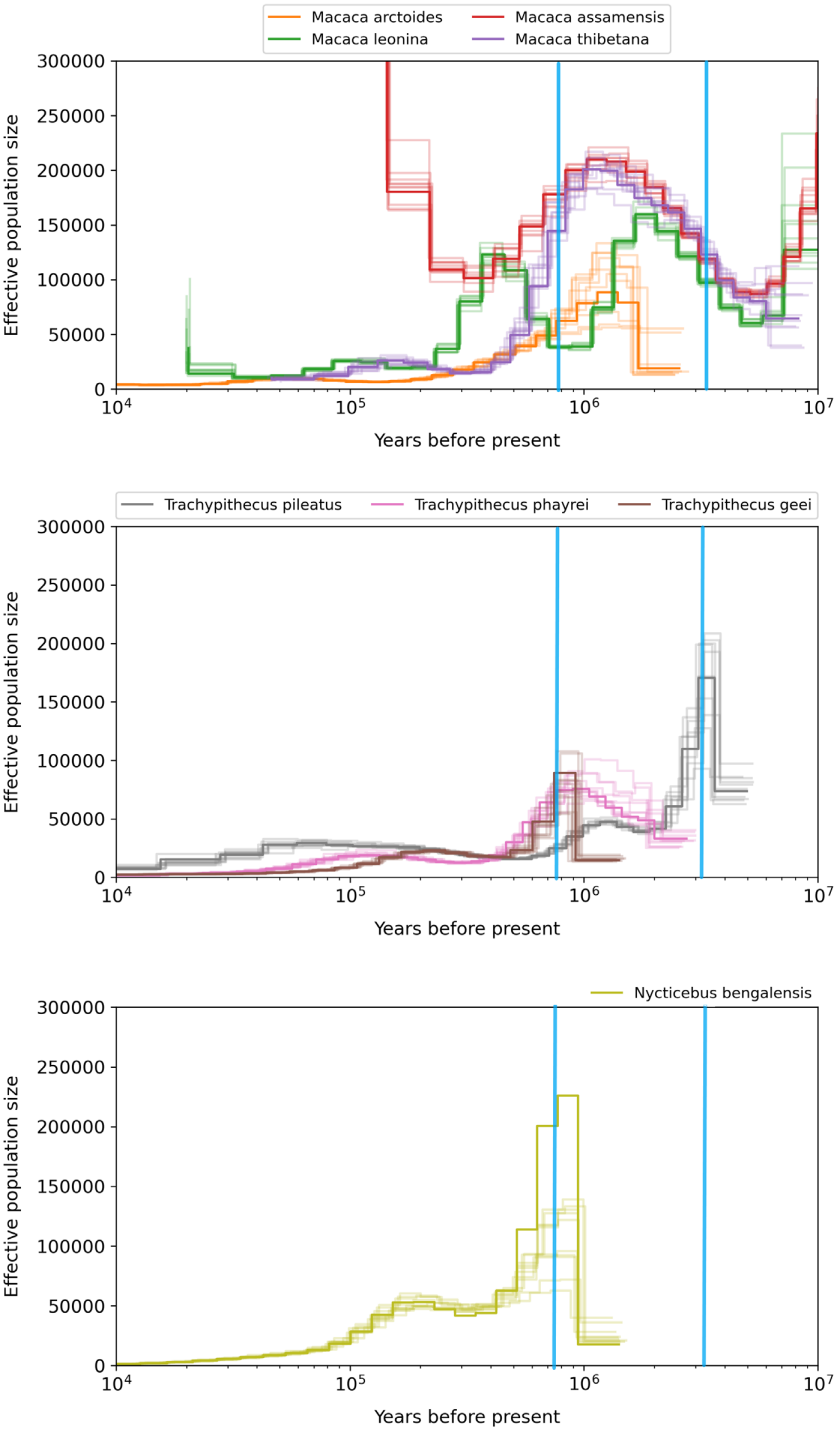
Demographic history of the *Macaca*, *Trachypithecus* and *Nycticebus bengalensis* , with perpendicular blue lines showing two SDM time points of, 3.3 million years ago (right) and 787,000 years ago (left). (A) All *Macaca* species show an increase between the time points of Pliocene and Pliestocene and then there is a stark decline towards the present. Also a clear divergence can be seen between *M. assamensis* and *M. thibetana* after the Pleistocene suggesting there evolutionary split. (B) The ancestor of *Trachypithecus pileatus* and *T. geei* has largest population size at about 3.3 million years. *T. geei* and *T. phayrei* have their respective peaks at 787 kya. (C) Sudden decline of *N. benghalensis* after 3.3 my marks reflects its vulnerability to the climatic fluctuations of the Pliestocene.

For all the species, we could get the estimates of effective population size till at least the beginning of Pleistocene. Some species data was also showing the estimates going into 10 million years ago, though we did not take them into consideration as climate data for such ancient time period is not available. There was high temporal variation in all the four *Macaca* species, with differing patterns of rise and decline in population sizes (Figure 1A). *M. assamensis* and *M. thibetana* have similar trajectories till about 800,000 years ago. We see a peak for effective population size in our *M. arctoides* MSMC plot at about 1.3–1.4 million years ago and then there is steady decline regardless of climatic conditions. In the case of M. leonine, there are two drastic population decline, one at 2 million years ago and another at 350,000 years ago. Among the langurs, *Trachypithecus pileatus* shows a rise at about 3.3 million years and then declines (Figure 1B). *T.geei* data shows a rise at mid‐Pleistocene and declines after that, this pattern is similar for *T. phayrei. Nycticebus bengalensis* also has the highest number of individuals at mid Pleistocene and since has declined (Figure 1C). The results were not very clear for and we have included it in the Supporting Information. *Hoolock hoolock* had equally large population comparative to recent times, with highest point at about 800,000 years ago and has declined since (Figure S1).

### 3.2 Maxent Models

The bioclimatic variables selected for each species are shown in Table S3. AUC test and training values for all the species are above 0.9 (Table S4), this indicates that the potential distribution of all the species fits well with the data. For most of the species, precipitation seems to be the highest contributing factor towards their predicted distribution, although temperature also governs the distribution of some of the primate species in northeast India (Table S4). By looking at the SDM outputs from Maxent analysis we can see that, except for *Hoolock hoolock* (Figure S1), the projected distribution for most species increased in the Pleistocene (~787 Ka) period as compared to what it was in the Pliocene (~3.3 Ma). Then finally in the current time period (1979–2013), we can see that the potential distribution of most of the species decreased as compared to their distribution during the Pleistocene. There were exceptions to this result, the potential distribution of *T. phayrei* and *N. bengalensis* shows a slight increase as compared to their distribution during Pleistocene, and the distribution of *H. hoolock* doesn't show any change between the current and the Pleistocene.

## 4 Discussion

In our analysis, we found that the effective population size of all the primate species have decreased with time towards the present. On the contrary, the distribution models of different species showed different variations, with some contracting their distributions and other expanding it. This should be due to their individual genera's and species' evolutionary trajectories. We have detailed the environmental response of each genus below to provide clearer implications from the study.

### 4.1 Genus—*Macaca*

**FIGURE 2:**
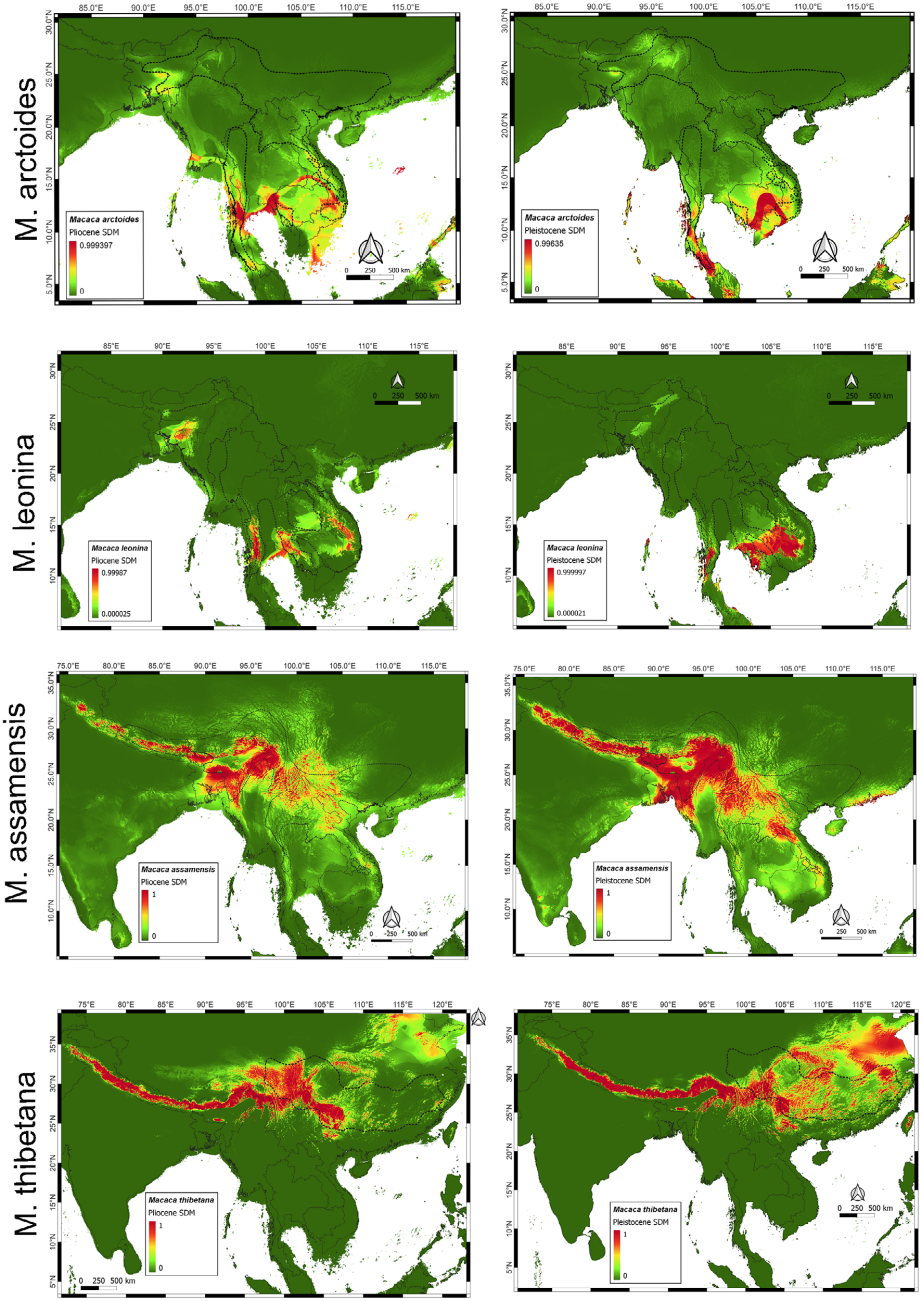
Pleistocene and Pliocene distribution maps for four species of the genus *Macaca*. It can be evinced that *M. assamensis* and *M. thibetana* has higher distribution probabilities in northern and western regions, and *M. leonina* and *M. arctoides* have higher probabilities in Indochina showing their where their populations could have evolved.

Among all the Macaca members in NE India, *M. assamensis* and *M. thibetana* both belong to the sinica group of macaques, along with *M. sinica* , *M. radiata* , *M. munzala* , *M. leucogenys* , and *M. sela* (Roos and Zinner 2015). MSMC2 plots of *M. assamensis* and *M. thibetana* reflect their genetic history, as they are phylogenetically closest species which have diverged as recently as 750,000 years ago (Roos et al. 2019). We can also observe the effective population sizes are identical till the mid‐Pleistocene boundary and then both the species diverge. Going back in the time, this should be due the difference in coalescences up till that point. Earlier to that point of time, the demographic history of the species seems identical. Therefore that specific point suggests speciation in the common ancestor (Schiffels and Durbin, 2014). The species distribution models show changes in the geographical distribution over time. The most suitable habitat for both these species in Pliocene (Figure 2) is the present‐day northeast India, with *M. thibetana* showing some distribution in the Himalayan foothills. By Pleistocene, the distribution of *M. assamensis* have gone towards the west, making its way into the Himalayan foothills. *M. thibetana* has taken the route in the northeast direction with Tibet and southern China as its spread, along with suitability shown in Nepal too, overlapping with *M. assamensis* . This becomes clearer as we come to present where the east–west distribution of *M. assamensis* and *M. thibetana* are distinct (Choudhury 2022; Khanal et al. 2018). Since the common point of divergence is northeastern India, we suggest it must be the location where the speciation could have taken place, between 700,000 and 800,000 years ago.

*M. arctoides* (Stump tailed macaque) is another unique species which has been variously classified as either the member of the fascicularis group, or mulatta group or as a sole member of its own arctoides group (Song et al. 2021). Its evolution has also been an issue of intense debate in the community (Fan et al. 2018). *M. arctoides* diverged before the split of *M. assamensis* and *M. thibetana* ; and later at 3.43 million years ago from the mulatta group (Li et al. 2009b; Roos et al. 2019). Observing the distribution models and population size plots, we can see that at all the three time points the population is confined to the mainland southeast Asia. Other relatives of *M. arctoides* are present at both the north and south of this distribution, like *M. cyclopis* in Taiwan and *M. fuscata* in Japan. It has also been strongly suggested that *M. arctoides* is a result of hybridization between a proto‐ arctoides and female *M. mulatta* (Fan et al. 2018). Taking into account all this evidence, we speculate that a proto‐ arctoides ancestor could have evolved in the Indochina region and then various populations would have proceeded to different regions, decreasing the effective population size at about 1.3–1.4 million years ago. Following this, a population might have moved northward and hybridized with *M. mulatta* to give rise to modern *M. arctoides* .

Another species we considered was *M. leonina* (Northern Pig tailed macaque). This is a sister species of *M. nemestrina* , the southern pig‐tailed macaque, which also lends its name to the nemestrina group, which includes all the Sulawesi macaque species and *M. silenus* (lion‐tailed macaque) in peninsular India. This is the oldest diverged group of macaques, after their initial split from *M. sylvanus* (Barbary macaque) (Roos et al. 2019). As with *M. arctoides* , the distribution models suggest that *M. leonina* also had suitable climates in southeast Asia, during Pliocene and Pleistocene, and later it must have expanded its range towards northeast India. Observing the MSMC plots, we expect that there could have been a speciation event at 2 mya point, as this is supported by the literature (Abdul‐Latiff and Md‐Zain 2021; Ram et al. 2015). This could be the point when both southern and northern pig‐tailed macaque species split somewhere in Indochina, with proto‐*leonina* continuing its march to the north. Another splitting event, the most recent one, could have taken place during Pleistocene glaciations, which would have given the modern species of *M. leonina* and *M. silenus* . *M. silenus* had formed from a population of *M. leonina* which got isolated during glaciations and went towards peninsular India in the search of more conducive environments. After the Pleistocene, the contact between both the population was lost due to lack of continuous forests, followed by the allopatric speciation of *M. silenus* (Ram et al. 2015; Singh et al. 2002). A similar study of *M. silenus* would be immensely beneficial to support or refute these conjectures.

### 4.2 Genus—Trachypithecus

**FIGURE 3:**
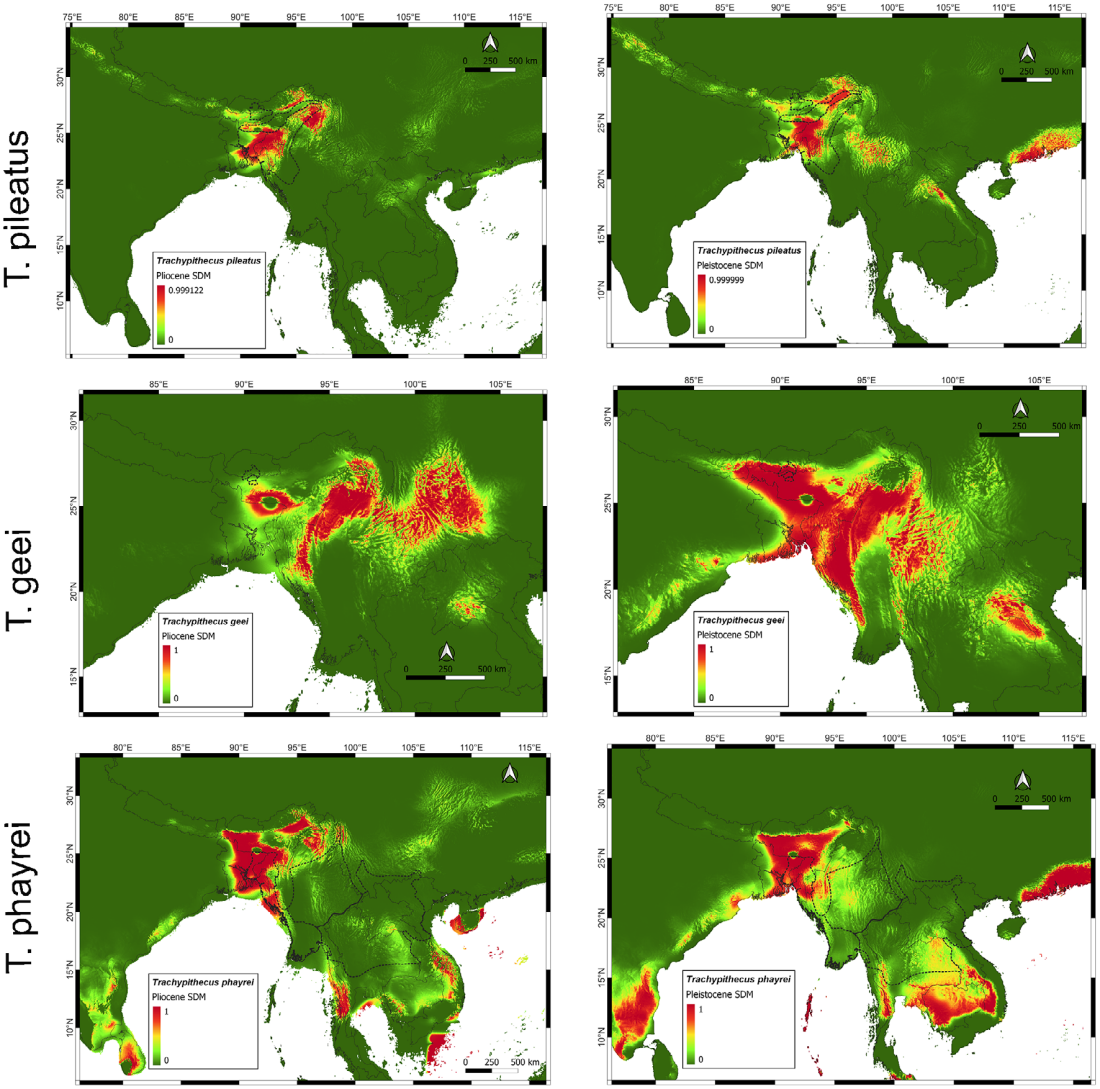
Pleistocene and Pliocene distribution maps for three species of the genus *Trachypithecus*. As can be seen that the distribution probabilities are confined to the northeast Indian region, with slight extension of *T. geei* towards the east. Combined with the demography data, we can speculate that northeast region could have been the region of speciation for *Trachypithecus*.

*Trachypithecus* are leaf monkeys which are related to more common hanuman langurs from the genus Semnopithecus. *Trachypithecus* consists of about 16 species, out of which three are found in northeast India: *Trachypithecus pileatus* (capped langur), *T. geei* (golden langur) and *T. phayrei* (Phayre's leaf monkey) (He et al. 2012; Roos et al. 2020; Wang et al. 2012). We were able to get samples and location data for all the three species and analyzed demography and historical distribution for them (Figure 3).

Though there is a lack of a conclusive phylogeny, mitochondrial data has shown that the ancestor of *T. pileatus* and *T. geei* was the first one to split in the *Trachypithecus* genus at about 4.5 million years ago (Roos et al. 2020). The divergence between *T. pileatus* and *T. geei* is much recent, at about 500,000–800,000 years ago. We find a peak in the effective population size of *T. pileatus* exactly at the 3.3 million years, showing that this species preferred warmer climate of mid‐ Pliocene. There is a drastic decline as the Pleistocene glaciations start, only to rise and then again declining at 787 kya mark. Here, we expect the divergence of *T. geei* from *T. phayrei* . A corroboration also comes from the demography of *T. geei* . Here the peak is at the 787 kya point, where the *T. pileatus* population goes in decline, suggesting that some population might have speciated to *T. geei* during this period.

The distribution map shows extended range of the putative ancestral population of *T. pileatus* and *T. geei* towards Myanmar in mid‐ Pleistocene. From there, some population of (or related) to *T. pileatus* could have seek refugia in the hills of present‐day Assam‐Bhutan border and then have evolved into *T. geei* . Hybridization between Hanuman langurs (Semnopithecus spp.) and *T. pileatus* has also been proposed as a reason for the speciation of *T. geei* as the present distribution of the species falls into hybridization zone of both Hanuman langurs and capped langur (Arekar, Parigi, and Karanth 2021).

*T. phayrei* is a member of a different group, obscurus, within the lutungs which have recently diverged into four species. The split of *T. phayrei* from its ancestral group has happened at 1.4 million years ago according to the mitochondrial data (Roos et al. 2020). In the distribution maps, in both Pliocene and Pleistocene, the population in all the scenarios seems to always concentrated in northeast India and Bangladesh for *T. phayrei* . In our demographic estimates, we find the peak population at 1‐million‐year mark, which then steadily declines after mid‐ Pleistocene. After the split, *T. phayrei* looks to have survived the ice ages with no further divergence in new species. If we look at historical distribution maps, it is evident that *T. phayrei* has had a constant ancestral distribution in northeast India and Bangladesh, and however later, it started dispersing towards southeast Asia as seen in the present distribution map (Figure S2).

Seeing the pattern of the three *Trachypithecus* species in northeast India, we can hypothesize that the region can be a ‘centre of origin’ for the ancestral *Trachypithecus* species and might also be for a proto‐ *Trachypithecus‐Semnopithecus* species. This idea has also been suggested by some other authors based on molecular work (Karanth 2010; Osterholz, Walter, and Roos 2008; Roos et al. 2011).

### 4.3 Genus—Nycticebus

**FIGURE 4:**
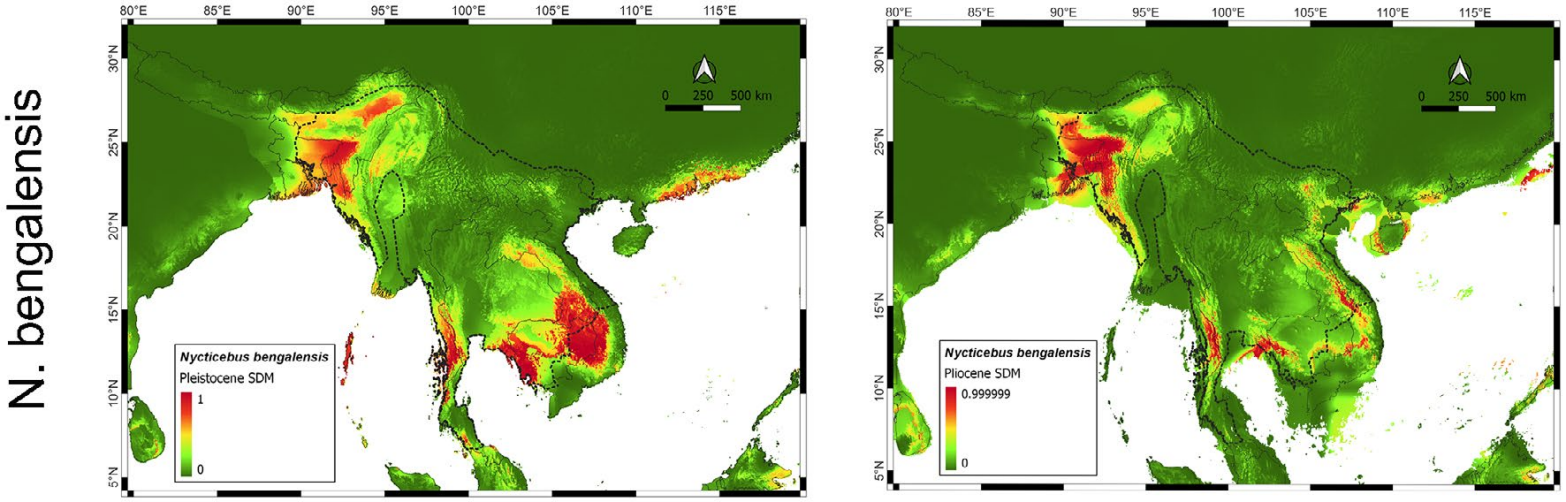
Pleistocene and Pliocene distribution maps for the loris species, *Nycticebus bengalensis*. The distribution is disjoint in this case with primary concentrations in northeast India in both the periods and then towards the tip of Indochina peninsula at 787 kya time point.

*Nycticebus* is the genus of slow lorises, which contain eight species, distributed all over the southeast Asia. *Nycticebus bengalensis* is the single species found in northeast India and also the one with western most distribution in the genus. *Nycticebus* are the oldest genus among lorisids and their ancestor can be traced back till Miocene. The earliest lineage of *N. pygmaeus* diverged at 11 million years ago (Chen et al. 2006; Munds et al. 2018). According to the mitochondrial analysis, *N. bengalensis* is one of the most recent lineages, with the last split from *N. javanicus* in Pleistocene. The lack of data for such old conditions prevents us from commenting anything about the evolution of the whole genus. Our distribution models indicate the probability of the presence of *N. bengalensis* in northeast India at both the time periods of Pliocene and Pleistocene (Figures 1C and 4). Observing the demographic history, we can say that *N. bengalensis* thrived in mid Pleistocene, and then suddenly declined as the glaciations proceeded. The reason for this decline should be constant variation in climate, as this highly specialized primate might be more sensitive to climatic fluctuations, with a lack of forests and less foraging opportunities (Nekaris 2014). We expect this sudden decline period can also be the time when the last split between *N. bengalensis* and *N. javanicus* could have happened, though this speculation has to be taken with a grain of salt. Present distributions of *N. bengalensis* and *N. javanicus* are not continuous, as the *N. coucang* lies in the middle of them. This disjoint distribution makes it difficult to guess any historical biogeographic scenario, especially when location and genetic data of most species in the genus are scarce. But our analysis strongly suggests that *N. bengalensis* or its ancestor had a suitable habitat in northeast India till at least Pliocene, and then it might have gone under population bottleneck and speciation during the repeated glacial periods of Pleistocene.

### 4.4 Genus—*Hoolock*

Gibbons are the small apes, among which Hoolock is the genus found in India, with *Hoolock hoolock* (Western hoolock gibbon) as its sole representative. Gibbon diverged from great apes at about 16–17 million years ago and then the radiation of gibbon family into four genera began at ~6 million years ago (Carbone et al. 2014; Trivedi et al. 2021). *Hylobatidae* phylogeny is still a matter of debate and is pending to be resolved even after extensive research using various datasets and techniques (Carbone, Okhovat, and Roos 2023). In our demographic and distribution history, the conclusions seem to be marred by these confusions about gibbon evolution. The effective population size of *H. hoolock* has a small plateaued peak at mid‐ Pliocene and then a steep rise in population after mid‐ Pleistocene followed by a drastic decline (Figure S1). Therefore, the warmer period of Pliocene has suited the gibbons, but increased oscillations of Pleistocene in comparatively recent history seem to be averse to their preference of habitat. Observing the distribution models also, the hotter color in Pleistocene shows higher potential distribution probabilities than Pliocene, though the area remains almost similar. There is a small distribution in the areas of southern Assam, Meghalaya and Bangladesh. Another place of distribution is in the upper Assam. It is shown as disjoint by the model. As the time has gone by, there is a slight decrease in the southern distribution and a slight increase in the northern one. This change in the distribution cannot be termed as significant enough to give strong conclusions about the *H. hoolock* evolutionary history.

In summary, our findings suggest that primates in the northeast India have followed variety of evolutionary trajectories to become the confluence of various species present today. This also supports our hypothesis, that there is a stark drop in the effective population sizes of all the studied species. This can or cannot be due to anthropogenic factors and this is an avenue which should be explored further with higher resolution analysis. But a few genomes can only give this much and better population sampling is needed in the future. Our analyses has limitations regarding the paucity of geographical data as well as availability of more samples from each species. With more dispersed sampling, population level variance will also provide newer insights in diversity and ultimate reason of it, in the region. Similarly, field records about the presence of species will give better resolution to the distribution modeling inputs. Our present investigation is an initial study and paves way for such more detailed analyses in the northeast India, which suffers from lack of such investigations. Keeping this mind, we strongly emphasize the need for an extensive future research programme to understand primates and other taxa, which will consecutively assist in better conservation of the region and its biodiversity.

## Author Contributions

**Mihir Trivedi:** conceptualization (equal), data curation (equal), formal analysis (equal), investigation (equal), methodology (equal), validation (equal), writing – original draft (equal), writing – review and editing (equal). **Shivakumara Manu:** formal analysis (equal), investigation (equal), methodology (equal), software (equal), validation (equal), writing – original draft (equal). **Kunal Arekar:** data curation (equal), formal analysis (equal), investigation (equal), methodology (equal), writing – original draft (equal). **Lukas F. K. Kuderna:** data curation (equal), formal analysis (equal), methodology (equal), resources (equal), writing – review and editing (equal). **Jeffrey Rogers:** data curation (equal), formal analysis (equal), resources (equal), writing – review and editing (equal). **Kyle Kai‐How Farh:** data curation (equal), funding acquisition (equal), methodology (equal), resources (equal), writing – review and editing (equal). **Tomas Marques Bonet:** funding acquisition (equal), project administration (equal), resources (equal), supervision (equal), writing – review and editing (equal). **Govindhaswamy Umapathy:** conceptualization (lead), funding acquisition (lead), project administration (lead), resources (lead), writing – review and editing (lead).

## Conflicts of Interest

All authors declare no conflicts of interest except Kyle Kai‐How Farh and Lukas F. K. Kuderna are employee of Illumina.

## Supporting information


Data S1.

## Data Availability

The DNA sequences used for the analysis is deposited in the European Nucleotide Archive under the accession number PRJEB49549. The geographical coordinates for the locations of the concerned species were taken from the GBIF (Global Biodiversity Information Facility, https://www.gbif.org/dataset/search?type=OCCURRENCE) database.
