# Supplementary material for: Historical Demography and Species Distribution Models Shed Light on Speciation in Primates of Northeast India
Source: Ecol Evol. 2025 Feb 25;15(2):e70968. doi: 10.1002/ece3.70968 (PMC11850985; doi:10.1002/ece3.70968)
Supplement: Supplementary file 1 — Data S1. [file ECE3-15-e70968-s001.docx]

SUPPLEMENTARY FIGURES AND TABLES


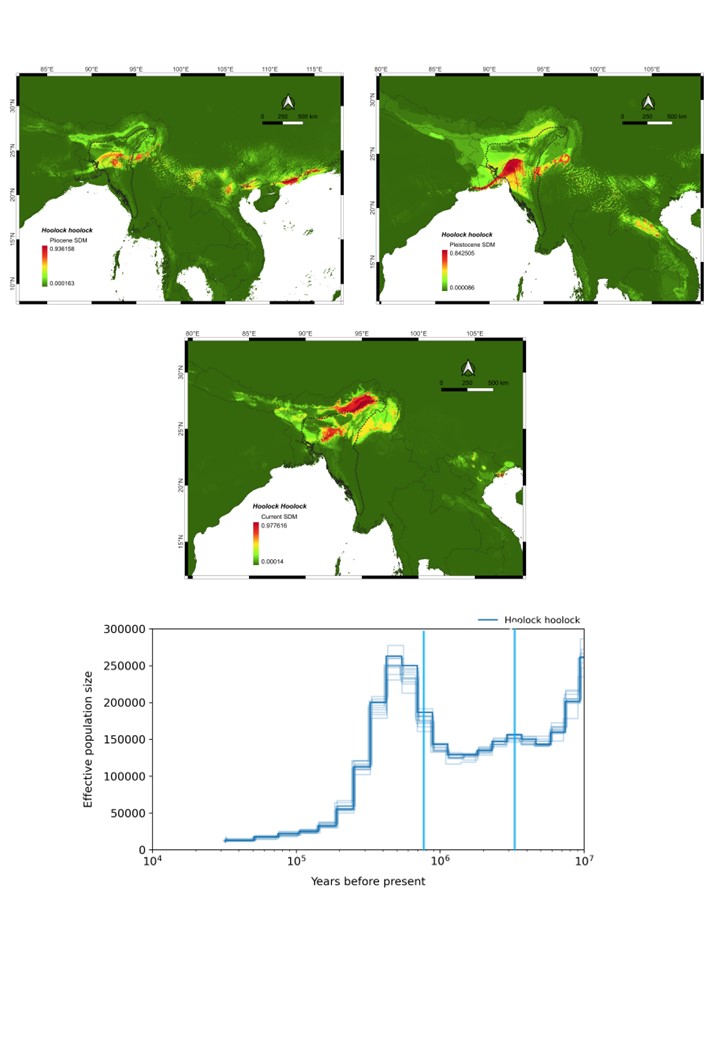


Figure S1: Distribution models and demographic history of *Hoolock hoolock.* In the distribution models, the gibbon species is predicted to be only in small regions of the northeast India.


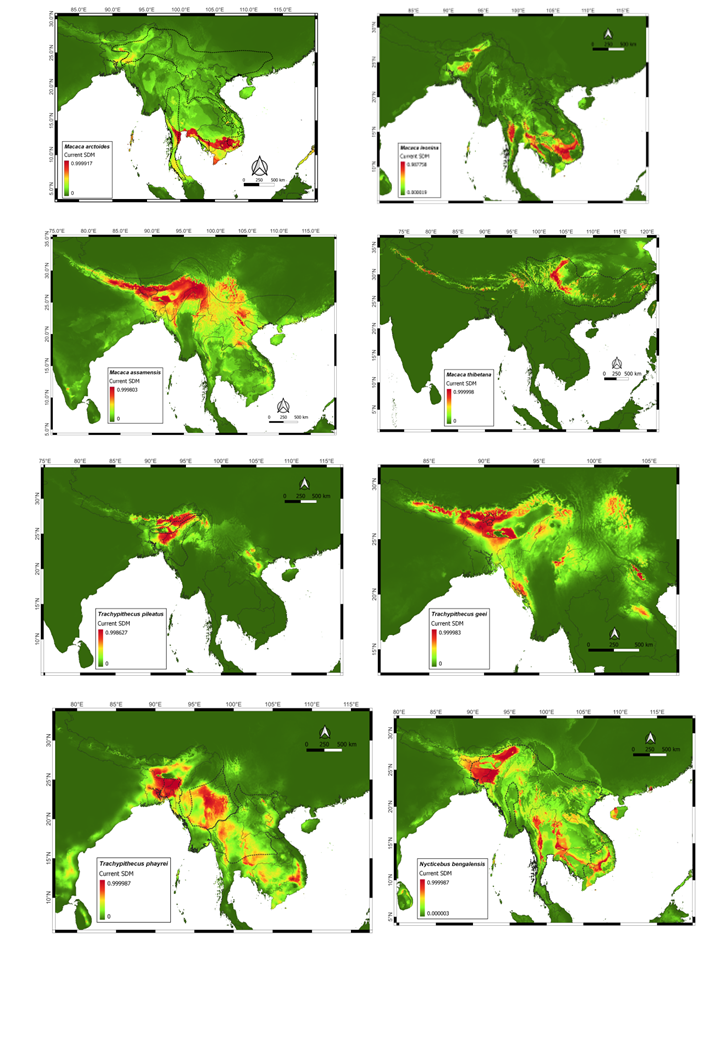


Figure S2: Distribution models for present era for all the nine species

Table S1: Mutation rate and generation time used by Ne estimation

| S. No. | Species | Mutation rate | Generation time | Reference |
| --- | --- | --- | --- | --- |
| 1 | *Macaca arctoides* | 5.80E-09 | 13 | (R. J. Wang et al., 2020) |
| 2 | *M. assamensis* | 5.80E-09 | 12 | (R. J. Wang et al., 2020) |
| 3 | *M. thibetana* | 5.80E-09 | 12 | (R. J. Wang et al., 2020) |
| 4 | *M. leonina* | 5.80E-09 | 13 | (R. J. Wang et al., 2020) |
| 5 | *Trachypithecus phayrei* | 1.36E-08 | 12 | (Zhou et al., 2014) |
| 6 | *T. pileatus* | 1.36E-08 | 12 | (Zhou et al., 2014) |
| 7 | *T. geei* | 1.36E-08 | 12 | (Zhou et al., 2014) |
| 8 | *Hoolock hoolock* | 1.00E-09 | 15 | (Veeramah et al., 2015) |
| 9 | *Nycticebus bengalensis* | 1.52E-08 | 8 | (Campbell et al., 2021) |

Table S2: Feature class type and RM values selected for the 9 species after the model selection analysis. FC = Feature class; RM = Regularisation multiplier

| Species | FC | RM |
| --- | --- | --- |
| *Hoolock hoolock* | LQPT | 1 |
| *Macaca arctoides* | LQPT | 1 |
| *Macaca assamensis* | QPT | 2 |
| *Macaca leonina* | T | 1 |
| *Macaca thibetana* | QPT | 1 |
| *Nycticebus bengalensis* | QPT | 1 |
| *Trachypithecus geei* | LQ | 0.5 |
| *Trachypithecus phayrei* | LQP | 0.5 |
| *Trachypithecus pileatus* | QPTH | 1 |

Table S3: Bioclimatic variables selected for each of the 9 species for Palaeodistribution modelling in Maxent.

| *M. arctoides* | *M. assamensis* | *M. leonina* | *M. thibetana* | *T. geei* | *T. phayrei* | *T. pileatus* | *N. bengalensis* | *H. hoolock* |
| --- | --- | --- | --- | --- | --- | --- | --- | --- |
| bio1 | bio1 | bio1 | bio4 | bio1 | bio1 | bio1 | bio1 | bio1 |
| bio4 | bio4 | bio4 | bio11 | bio4 | bio4 | bio4 | bio4 | bio4 |
| bio12 | bio13 | bio12 | bio12 | bio13 | bio12 | bio13 | bio12 | bio12 |
| bio14 | bio15 | bio14 | bio14 | bio15 | bio14 | bio14 | bio14 | bio15 |
| bio15 | bio17 | bio15 | bio15 | bio17 | bio15 | bio15 | bio15 | bio17 |
| bio18 | bio18 | bio18 | bio18 | bio18 | bio18 | bio18 | bio18 | bio18 |

H = *Hoolock*, M = *Macaca*, N = *Nycticebus*, T = *Trachypithecus*

Table S4: AUC test, AUC training and top three bioclimatic variables showing the highest contribution to the model.

| Species | AUC Test | AUC Training | Variable | Percent contribution | Permutation importance |
| --- | --- | --- | --- | --- | --- |
| *Hoolock hoolock* | 0.99 | 0.993 | bio18.tif | 64.8 | 1.7 |
|  |  |  | bio4.tif | 13.5 | 21.1 |
|  |  |  | bio1.tif | 6.9 | 57.1 |
| *Macaca arctoides* | 0.951 | 0.975 | bio4.tif | 65 | 76.3 |
|  |  |  | bio14.tif | 16.7 | 8.2 |
|  |  |  | bio15.tif | 9.5 | 4.5 |
| *Macaca assamensis* | 0.954 | 0.96 | bio4.tif | 42.9 | 44.6 |
|  |  |  | bio18.tif | 22.6 | 2.2 |
|  |  |  | bio17.tif | 14.2 | 26.6 |
| *Macaca leonina* | 0.98 | 0.988 | bio15.tif | 37.1 | 54.5 |
|  |  |  | bio4.tif | 34 | 15.4 |
|  |  |  | bio12.tif | 10.1 | 8.6 |
| *Macaca thibetana* | 0.988 | 0.992 | bio11.tif | 48 | 82.1 |
|  |  |  | bio4.tif | 27.6 | 9.3 |
|  |  |  | bio18.tif | 14.5 | 7.6 |
| *Nycticebus bengalensis* | 0.911 | 0.98 | bio15.tif | 34.7 | 36.7 |
|  |  |  | bio18.tif | 23.9 | 6.5 |
|  |  |  | bio4.tif | 22.2 | 33.4 |
| *Trachypithecus geei* | 0.989 | 0.99 | bio18.tif | 48.2 | 0.9 |
|  |  |  | bio4.tif | 21.4 | 41.5 |
|  |  |  | bio17.tif | 11.8 | 37.8 |
| *Trachypithecus phayrei* | 0.958 | 0.962 | bio4.tif | 35.1 | 19.5 |
|  |  |  | bio14.tif | 20.3 | 43.2 |
|  |  |  | bio15.tif | 20.3 | 21.6 |
| *Trachypithecus pileatus* | 0.987 | 0.992 | bio18.tif | 55.4 | 0.7 |
|  |  |  | bio4.tif | 28.6 | 29.2 |
|  |  |  | bio15.tif | 8.3 | 23.8 |

Table S5: The 19 bioclimatic variables with codes and full names

| Bioclimatic Variable code | Variable name |
| --- | --- |
| Bio_1 | Annual Mean Temperature [°C*10] |
| Bio_2 | Mean Diurnal Range [°C] |
| Bio_3 | Isothermality [Bio_2/Bio_7] |
| Bio_4 | Temperature Seasonality [standard deviation*100] |
| Bio_5 | Max Temperature of Warmest Month [°C*10] |
| Bio_6 | Min Temperature of Coldest Month [°C*10] |
| Bio_7 | Temperature Annual Range [°C*10] |
| Bio_8 | Mean Temperature of Wettest Quarter [°C*10] |
| Bio_9 | Mean Temperature of Driest Quarter [°C*10] |
| Bio_10 | Mean Temperature of Warmest Quarter [°C*10] |
| Bio_11 | Mean Temperature of Coldest Quarter [°C*10] |
| Bio_12 | Annual Precipitation [mm/year] |
| Bio_13 | Precipitation of Wettest Month [mm/month] |
| Bio_14 | Precipitation of Driest Month [mm/month] |
| Bio_15 | Precipitation Seasonality [coefficient of variation] |
| Bio_16 | Precipitation of Wettest Quarter [mm/quarter] |
| Bio_17 | Precipitation of Driest Quarter [mm/quarter] |
| Bio_18 | Precipitation of Warmest Quarter [mm/quarter] |
| Bio_19 | Precipitation of Coldest Quarter [mm/quarter] |

**References for the downloaded location data from GBIF for each species**

GBIF.org (*Hoolock hoolock*) (30 April 2022) <https://doi.org/10.15468/dl.8jy8u8>

GBIF.org (*Macaca arctoides*) (30 April 2022) <https://doi.org/10.15468/dl.593s7e>

GBIF.org (*Macaca assamensis*) (30 April 2022) <https://doi.org/10.15468/dl.cbz3h4>

GBIF.org (*Macaca leonina*) (30 April 2022) <https://doi.org/10.15468/dl.crmerr>

GBIF.org (*Macaca thibetana*) (30 April 2022) <https://doi.org/10.15468/dl.5aerz4>

GBIF.org (*Nycticebus bengalensis*) (30 April 2022) <https://doi.org/10.15468/dl.b5mw2w>

GBIF.org (*Trachypithecus geei*) (30 April 2022) <https://doi.org/10.15468/dl.nhqh5c>

GBIF.org (*Trachypithecus phayrei*) (30 April 2022) <https://doi.org/10.15468/dl.x89292>

GBIF.org (*Trachypithecus pileatus*) (30 April 2022) <https://doi.org/10.15468/dl.7cadqh>
